# Triggering Drug Release and Thermal-Disrupting Interface Induced Mitigation of Composite Photothermal Hydrogel Treating Infectious Wounds

**DOI:** 10.3389/fbioe.2021.796602

**Published:** 2021-12-13

**Authors:** Long Hua, Hu Qian, Ting Lei, Wenbin Liu, Xi He, Yihe Hu, Pengfei Lei

**Affiliations:** ^1^ Department of Orthopedics, The First Affiliated Hospital, Medical College of Zhejiang University, Hangzhou, China; ^2^ Department of Orthopedics, Xiangya Hospital Central South University, Hunan Engineering Research Center of Biomedical Metal and Ceramic Implants, Changsha, China; ^3^ The Sixth Affiliated Hospital, Xinjiang Medical University, Urumqi, China

**Keywords:** triggering drug release, thermal-disrupting interface induced mitigation, tannic acid, Prussian blue, photothermal hydrogel

## Abstract

**Introduction:** With the development of photothermal technology, the appearance of composite photothermal hydrogels has increased the selectivity of treating infectious skin defects. However, how to design composite photothermal hydrogel with better antibacterial performance, reduce the resistance rate of bacteria, and the damage rate of normal tissue still needs further study.

**Methods:** The Prussian blue and tannic acid were loaded on polyacrylamide hydrogels. Characterization of DLS, Zeta potential, UV absorption spectrum, hydrogel swelling rate, scanning electronic microscopic, drug release profile, photothermal properties, *in vitro* cytocompatibility, and antibacterial properties. Experiments were measured by skin defect repair, antibacterial detection, and histological staining experiments.

**Results:** The polyacrylamide hydrogel with photothermal effect and controllable release of tannic acid was successfully prepared. The hydrogel has strong light transmittance and adhesion, and the swelling rate can reach 600%, which improves the self-cleaning ability. SEM results showed the porous structure of hydrogels, promoting cell growth. Through photothermal switches, the composite hydrogel represented adjustable and controllable drug release ability. Combined with the synergistic antibacterial effect of tannic acid, this further enhanced the antibacterial ability and reduced the probability of antibiotic resistance. The *in vitro* and *in vivo* experiments showed the hydrogel had good biocompatibility and excellent antibacterial properties, which could promote the repair of infectious skin defects in SD rats.

**Conclusion:** We fabricated a hydrogel with a triggering drug release rate, alleviating heat damage, transparent morphology, mechanical stability, strong adhesion, good biocompatibility, and synergistic antibacterial ability, which presents new treatment options for infectious skin defect repair.

## Introduction

Globally, one-third of all deaths are caused by infections (Li et al., 2018). Among them, the treatment of infectious skin defects due to acute injuries, burns, and chronic diseases such as diabetes remains a challenge (Ning et al., 2014; Zheng et al., 2018). Traditional treatments involve the use of drugs such as antibiotics. However, increasing drug resistance has reduced the effectiveness of this kind of treatment to near useless (Dai et al., 2017). At the same time, the loss of skin tissue reduces the ability to form a barrier against bacteria, which can exacerbate the infection (Mao et al., 2018). Therefore, there is an urgent need for a new treatment strategy to improve the current severe situation of inadequate treatment options.

With the development of photothermal technology, more and more photothermal nanomaterials have been used in the treatment of infectious skin defects because of their good photothermal conversion and bactericidal properties. Among them, metallic and nonmetallic nanoparticles are included such as Ag-based (Cao et al., 2020), Au-based (Ngo-Duc et al., 2020), Cu-based (Yu et al., 2020), and graphene-based NPs (Zhang et al., 2019). However, the application of these materials has been hindered due to their biotoxicity and instability, as well as the high temperature generated at the interface of biological tissues, which can cause damage to normal tissues (Hu et al., 2020). Therefore, the research and development of a composite material with low biotoxicity and good biocompatibility, which has excellent bactericidal performance and can reduce bacterial resistance to protect normal tissues, is still a goal pursued by scholars (Zhou et al., 2016).

The excellent stability and biocompatibility of metal-organic frameworks have been widely reported (Li et al., 2019). Among them, Prussian blue has been widely studied because of its simple synthesis method, strong structural stability, good biocompatibility, photothermal properties, and strong sterilization efficiency (Wang et al., 2018; Borzenkov et al., 2019; Cai et al., 2019; Li et al., 2019; Luo et al., 2019; Han et al., 2020; Mukherjee et al., 2020; Sharma et al., 2020) Maaoui et al. (2016). reported for the first time that Prussian blue nanoparticles could effectively kill *MRSA* and *E. coli* through the photothermal effect. Other literature reported that Prussian blue could kill *E. coli* 100% within 5 min through the photothermal effect. However, due to its high photothermal conversion rate, Prussian blue may increase the risk of damage to normal tissue (Jiang et al., 2018). Tannic acid is a polyphenolic compound, which is the active ingredient extracted from gallnut (Ninan et al., 2016; Yan et al., 2019; Liu et al., 2020). It has been reported in the literature that it has antibacterial, antioxidant, antiviral, and other activities. Tannic acid also has the function of inhibiting bacterial quorum sensing (QS) and preventing the formation of bacterial biofilms (Liu et al., 2020). However, tannic acid has limited antimicrobial ability against Gram-negative bacteria *E. coli*, which limits its application (Sahiner et al., 2016). Polyacrylamide has good biocompatibility and non-toxicity and is often used in drug delivery and skin repair research (Xue et al., 2019). Its 3D hydrogel network provides a moist environment for cells, promotes permeability of oxygen and water molecules, and protects against microbial invasion (Gan et al., 2018). Its good mechanical properties are characterized by strong stress and flexibility, so it is often possible to construct hydrogels with good properties without chemical modification (Chen et al., 2018; Fang et al., 2019). The electrostatic, hydrogen bonding, and hydrophobic interactions of the groups in hydrogel make it have the characteristics of strong viscosity, which can enhance the skin adhesion ability of the hydrogel (Xue et al., 2019; Hao et al., 2020). In addition, with the increase of water, the adhesion decreases, making the hydrogel easy to peel off (Ning et al., 2014). Therefore, the construction of polyacrylamide composite hydrogel loaded with Prussian blue nanoparticles and tannic acid is expected to improve its antibacterial performance and tissue repair ability while reducing the side effects of the material through synergistic effect.

**FIGURE 1:**
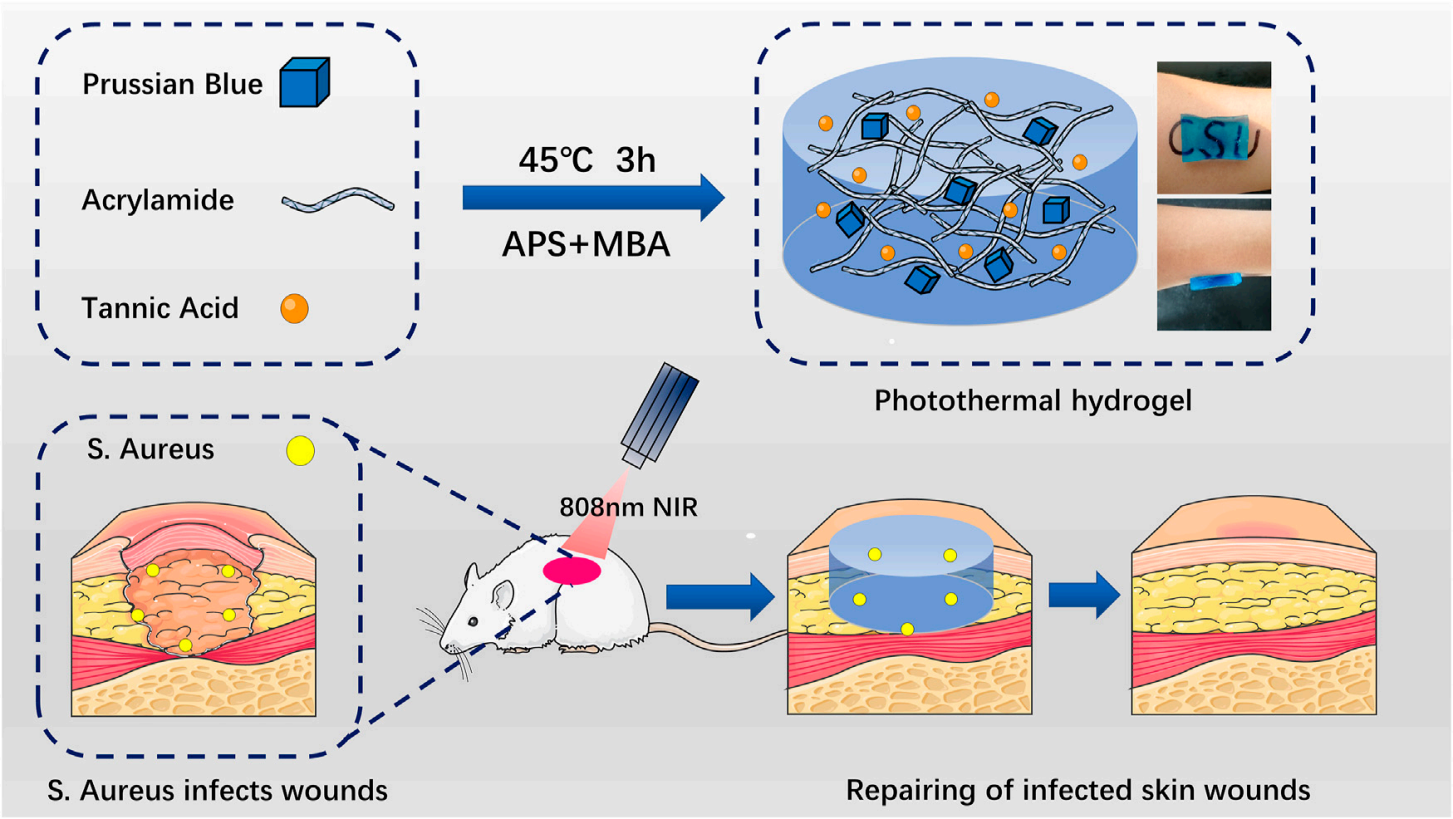
Schematic diagram of Prussian blue nanoparticles and tannic acid drug particles loaded on acrylamide hydrogel for the elimination of bacteria and promotion of skin repair through the synergistic photothermal and antibacterial action of the drugs. APS: ammonium persulfate. MBA: 4-sulfanylbutanimidamide.

In this study, polyacrylamide composite photothermal hydrogel containing Prussian blue nanoparticles and tannic acid was prepared by precipitation method and immersion method. We fabricate composite hydrogel, which has antimicrobial ability, skin repair ability, triggering drug release (TDR), and thermal-disrupting interface induced mitigation (TRIM) alleviating heat damage. In this paper, the physical and chemical characterization, *in vivo* and *in vitro* biocompatibility, anti-infection ability, and tissue repair ability of the composite photothermal hydrogel were detected and analyzed. The scientific hypothesis that this composite photothermal hydrogel has good biocompatibility and antibacterial ability to promote skin repair is put forward (Figure 1). There are no reports of the use of Prussian blue nanoparticles combined with tannic acid-loaded polyacrylamide composite photothermal hydrogel for the treatment of infectious skin defects.

## Results and Discussion

### Physical Characterization of Composite Photothermal Hydrogel

**FIGURE 2:**
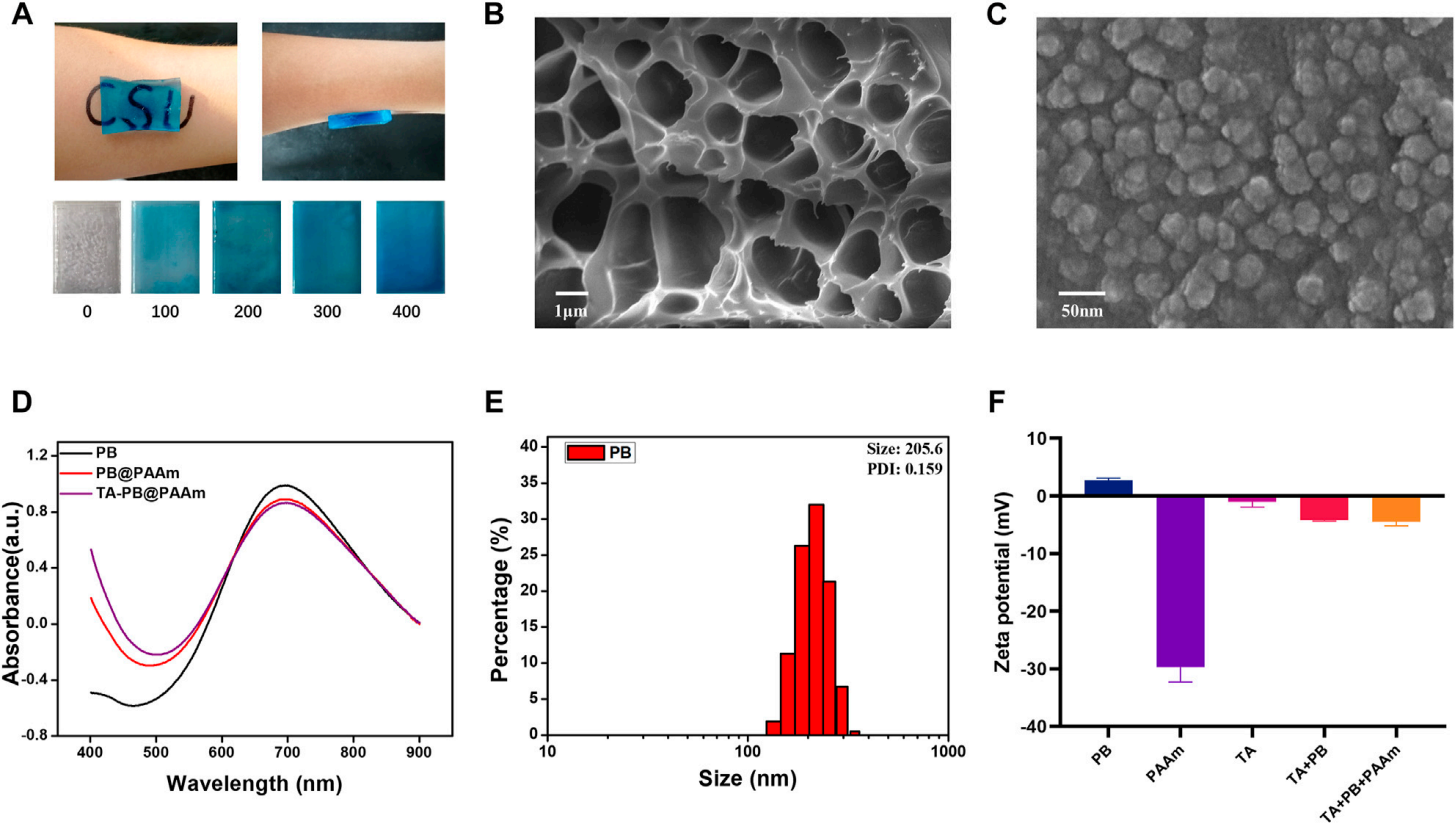
Physical and chemical characterization of hydrogel composites. **(A)** Appearance of the materials, which are prepared with different concentrations of Prussian blue and the adsorption properties (unit = ug/ml). **(B)** Porous structure of formed composite photothermal hydrogels. **(C)** SEM microscopic morphology of Prussian blue nanoparticles. **(D)** UV absorption spectra of PB, PB@PAAm and TA-PB@PAAm. **(E)** Particle size distribution of Prussian blue nanoparticles. **(F)** Zeta potential of PB shows positive charge.

In this study, TA-PB@PAAm hydrogel was successfully fabricated (Figure 2A). TA-PB@PAAm hydrogel presented a blue transparent peptone-like appearance, which was mainly due to the doping of Prussian blue nanoparticles. As the content of Prussian blue increased, the color of the hydrogel deepened. The hydrogel has a strong adhesion effect, which may be caused by electrostatic, hydrogen bonding, and hydrophobic interactions. According to the SEM test results (Figure 2B), TA-PB@PAAm hydrogel presented a uniformly distributed porous structure, and its pore size distribution was about 1 μm. SEM characterization of PB nanoparticles showed that they presented a cubic structure with a diameter of about 50 nm (Figure 2C).

The absorption peaks of PB, PB@PAAm, and TA-PB@PAAm were detected by UV-Spectrum, and it was found that their absorption peaks were 700 nm (Figure 2D). The particle size of PB, PB@PAAm hydrogel precursor solution, and TA-PB@PAAm hydrogel precursor solution were detected. The Zeta potential of PB, TA, TA + PB, PAAm hydrogel precursor solution, and TA-PB@PAAm hydrogel precursor solution were detected by particle size analyzer. The average particle size of PB was 205.6 nm and PDI was 0.156 (Figure 2E). Zeta potential results showed that the surface of PB was positively charged, TA was negatively charged, and finally the hydrogel was negatively charged (Figure 2F). The FTIR results are as follows. In the sample TA, the vibration peaks observed at 3402, 1716, and 1614cm^-1^ were respectively determined by the hydroxyl group, C=O in the carboxyl group and C-C in the benzene ring skeleton showed the presence of tannic acid. In sample PB, the vibration peaks caused by the metal -C≡N- metal at 2083 cm^-1^ and 594 cm^-1^ were observed, indicating the existence of PB. For the sample PAAm hydrogel, vibration peaks were not observed at 1,430 cm^-1^ and at 1,630 cm^-1^, indicating that the stretching vibration of the C=C bond after acrylamide cross-linking polymerization disappeared. At 2,980 cm^-1^ and 1,450 cm^-1^, the characteristic vibration peak was the C-H vibration peak which was in the -CH2- group. It was found that 1756 cm^-1^ represented the amide bond, 1,189 cm^-1^ represented the C-N bond, and 1,083 cm^-1^ represented the C-O-C bond. These characteristic vibration peaks indicated the formation of polyacrylamide. In addition to the characteristic vibration peak of PAAm, it could be observed that the vibration peak of the amide bond moved to 1,658 cm^-1^, and the vibration peak of C-O-C moved to 1,116 cm^-1^, which could be caused by the coordination effect between metal ions in Prussian blue and C-O functional groups in PAAm structure. For the TA-PB@PAAm sample, the characteristic vibration peak belonging to the benzene ring could be observed near 1,614 cm^-1^, which proved that TA structure also existed in the PB@PAAm structure (Supplementary Figure S1). The XRD results are as follows: For sample PB, only a wide diffraction peak was observed at 19.6°, proving that the Prussian blue structure synthesized was amorphous. For sample TA, only a wide diffraction peak was observed at 25.2°, proving that the TA was an amorphous structure (Supplementary Figure S2).

**FIGURE 3:**
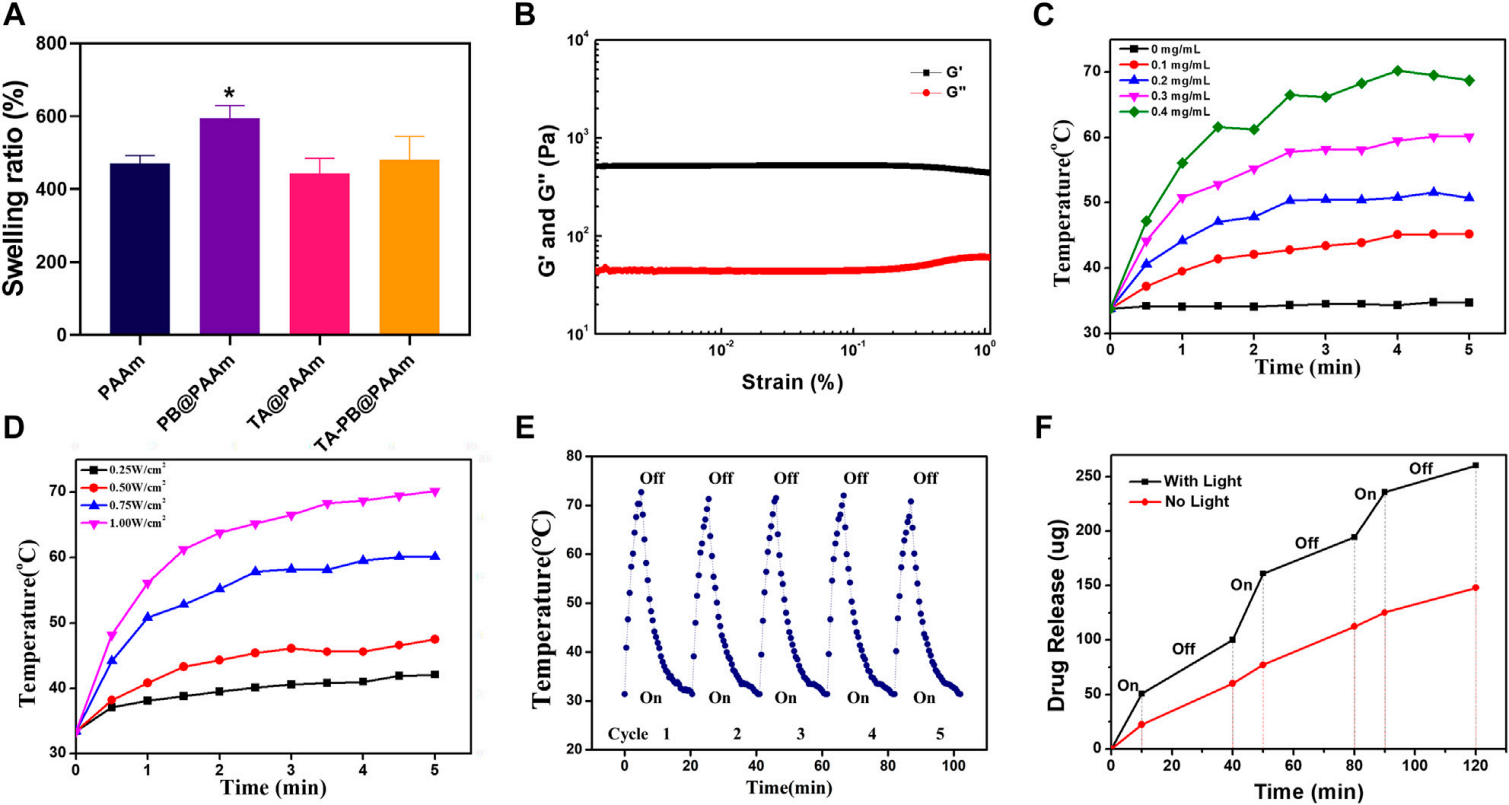
Physical and chemical characterization of TA-PB@PAAm composite hydrogels. **(A)** Swelling rate for different contents of composite photothermal hydrogels. **(B)** The preservation modulus G′ is always higher than the loss modulus G″, which proves that the material has good crosslinking performance. **(C)** Photothermal effect curve for different PB concentrations. **(D)** Photothermal effect curve under different illumination intensities. **(E)** Curve of temperature changes after cyclic illumination of the hydrogels. **(F)** Drug release curve under alternating light.

All the above results indicated that PB and PAAm were crosslinked and successfully loaded. We then loaded the hydrogels with different concentrations of TA by the immersion method and calculated the drug loading rates, which were 0.25,0.5, and 2.5%. With the increase of TA concentration, the drug loading rate of polyacrylamide also increases, so it has excellent drug loading capacity. Therefore, 5 mg/mL TA concentration was selected as the later application concentration according to the results. We calculated the swelling rate of PAAm, PB@PAAm, TA@PAAm, and TA-PB@PAAm, and they are 470.8 ± 17.5%, 594.1 ± 28.6%, 442.6 ± 33.8%, and 480.3 ± 52.4%, respectively. The composite photothermal hydrogel could absorb about 4–5 times its own mass of water, so it had a good water absorption capacity (Figure 3A). This excellent swelling ability can effectively absorb the exudated and exfoliated necrotic tissue cells from the wound during the treatment process, so it has the function of autolytic debridement.

The rheology was used to test the viscoelastic properties of the composite photothermal hydrogel. Storage modulus G′ of the hydrogel was always higher than loss modulus G″ by 500% strain, and G′ and G″ relatively represented pronounced plateau, indicating that the hydrogel could maintain a solid form and have a certain elasticity. This feature may be more suitable as a skin dressing (Van Den Bulcke et al., 2000) (Figure 3B).

### Photothermal Properties of Composite Photothermal Hydrogel

Different concentrations of PB loaded on hydrogels have different photothermal effects (Figure 3C). Among them, 0.4 mg/mL PB hydrogel had the strongest photothermal effect and could reach 68.7°C within 5 min. With the decrease of PB content, the photothermal effect of the hydrogel gradually weakened, and the maximum temperature of 0.1mg/mL PB was 45.2°C. Different illumination intensities of NIR-808 laser had different photothermal effects (Figure 3D). The PB hydrogel with 1W/cm2 had the strongest photothermal effect and could increase to 70.2°C within 5 min. With the decrease of illumination intensity, the photothermal effect of hydrogel gradually weakened, and the maximum temperature was less than 47.5°C under the illumination intensity of 0.5W/cm2. It has been reported that when the ambient temperature of cells exceeds 50°C, cell necrosis will occur. When the ambient temperature of cells is below 50°C and above 40°C, apoptosis occurs, but it can be repaired by human heat shock proteins (Yang et al., 2017). Therefore, we chose the parameters of PB concentration of 0.1mg/ml and illumination intensity of 0.5W/cm^2^ as the final photothermal conditions.

In order to test the reusability of the composite photothermal hydrogel, repeat irradiation was given. The temperature of the hydrogel reached its peak within 5 min. After the irradiation of NIR-808 was turned off, the temperature of the hydrogel dropped to the initial temperature within 15 min. With the increase of the number of cycles, the peak temperature and cycle time of hydrogel remain stable. It showed that the hydrogels had a good photothermal effect to ensure recycling and reuse (Figure 3E).

### *In Vitro* NIR Light-Triggered Drug Release

In order to calculate the release amount of tannic acid, we tested the absorbance value of tannic acid in the ultraviolet absorption spectrum, which showed the maximum absorption peak at 275 nm (Supplementary Figure S3). With the increase of the concentration of tannic acid, the absorbance value also increased. In order to prove that the drug would be released by setting the photothermal switch in a NIR-triggered “off-on way”, we measured the release profile of tannic acid by ultraviolet spectrum (Figure 3F). The TA-PB@PAAm hydrogel without NIR light had the ability of drug release in PBS solution, and the drug release amount slowly increased with a total release amount of 147.9 μg in 120 min. When the TA-PB@PAAm hydrogel was illuminated by NIR light, the drug release was significantly increased, and the total release reached 260.3 μg in 120 min. After the light was turned off, the drug release rate was the same as that without NIR light. The rate of drug release in the hydrogel changed as the light was switched on and off multiple times. It showed that the synergistic photothermal effect could promote the release of drugs in the hydrogel. NIR light acted as a switch in drug release, which might better promote the efficacy of hydrogel in the treatment of infection, and intelligently regulated the release rate of the drug, which was 1.76 times higher than that of the non-light group.

### Cell Compatibility of NIH-3T3 With Composite Photothermal Hydrogel

**FIGURE 4:**
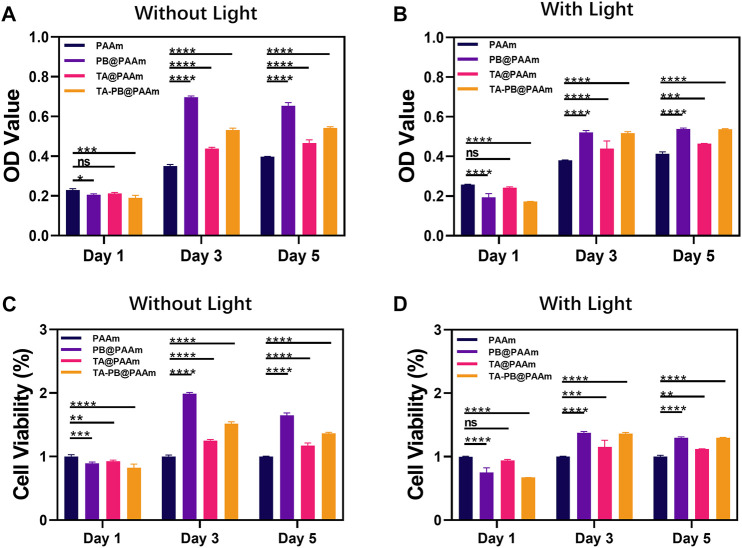
CCK-8 results of NIH-3T3 cells co-cultured with composite photothermal hydrogels at different time points. **(A–B)** Absorbance in PAAm, PB@PAAm, TA@PAAm, TA-PB@PAAm with or without light. **(C–D)** Survival rate in PAAm, PB@PAAm, TA@PAAm, TA-PB@PAAm with or without light. Data are mean ± SD (n = 3, *p < 0.05, **p < 0.01, ***p < 0.001, ****p < 0.0001, ns = no statistical difference).

Firstly, we measured the 50% lethality of tannic acid and Prussian blue to cells **(** Supplementary Figure S4 **)**. The 50% lethal dose of tannic acid in NIH-3T3 cells was 200 μg/ml and the 50% lethal dose of PB was 100 μg/ml. After that, we evaluated the cell compatibility in different hydrogels at different time points (1, 3, 5 days) by CCK-8 assay **(** Figures 4A–D **)**. With the increase of culture time, cells in each group showed a steady growth trend. On day one, the absorbance of the control group was higher than that of the other three experiment groups (PB@PAAm, TA@PAAm, and TA-PB@PAAm) both in the non-light and light conditions (non-light: 0.230, 0.206, 0.212, 0.190; light: 0.258, 0.194, 0.242, 0.173). On day three, the CCK8 levels of PB@PAAm, TA@PAAm and TA-PB@PAAm both in the non-light and light conditions were significantly higher than those of the blank control group (non-light: 0.350, 0.696, 0.438, 0.531; light: 0.380, 0.522, 0.439, 0.517). On day five, the CCK8 of PB@PAAm, TA@PAAm, and TA-PB@PAAm both in the non-light and light conditions were significantly higher than that of the blank control group, but the increasing rate was lower than that of the third day (non-light: 0.397, 0.654, 0.466, 0.542; light: 0.414, 0.538, 0.464, 0.536). These results indicate that our composite photothermal hydrogel has good cytocompatibility and can support cell adhesion and growth after implantation. The result may be a slow release of the drug due to the encapsulation of the hydrogel, thus reducing the damage to the cells.

### *In Vitro* Bactericidal Test of Composite Photothermal Hydrogel

**FIGURE 5:**
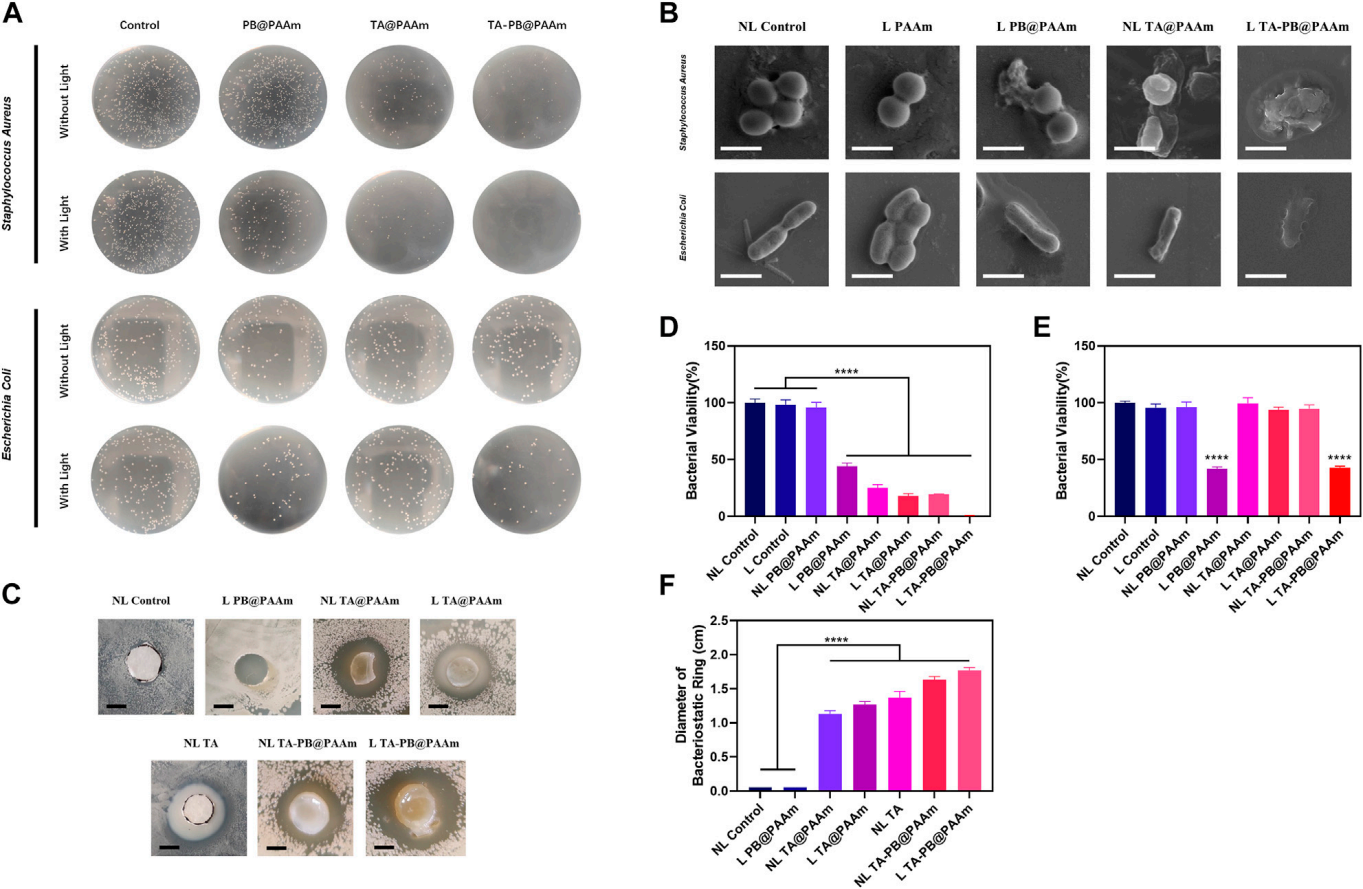
*In-vitro* bactericidal test of composite photothermal hydrogel. **(A)** Bacteriostatic experiment of different hydrogels on *Staphylococcus aureus* and *Escherichia coli*. **(B)** The SEM result of the bacterial morphology through the treatment of different hydrogels on *S. aureus* and *E. coli*. Scale bar = 1 μm. **(C)** Bacterial inhibition ring test results of different materials. Scale bar = 5 mm. **(D)** Quantitative results of the survival rate of *S. aureus*. **(E)** Quantitative results of survival rate of *E. coli*. **(F)** Quantitative results of the diameters of bacterial inhibition ring tests. Data are mean ± SD (n = 3, ****p < 0.0001).

We co-cultured *S. aureus* and *E. coli* with different kinds of hydrogels then inoculated the diluent solutions on the surface of the TSA plate for counting and evaluated the bactericidal effect of hydrogels. The survival rate of *S. aureus* was as follows (Figure 5A, D), NL control, L control, NL PB@PAAm, L PB@PAAm, N TA@PAAm, L TA@PAAm, NL TA-PB@PAAm, and L TA-PB@PAAm was 100, 98.5, 95.7, 44.2, 24.9, 17.9, 19.2, and 0.3%, respectively. Compared with the control group, the bacterial colony number of *S. aureus* inoculated on the surface of NL PB@PAAm was not significantly different, while the bacterial colony number of *S. aureus* inoculated on the surface of L PB@PAAm was significantly decreased. In addition, N TA@PAAm, TA@PAAm, and NL TA-PB@PAAm showed good bactericidal ability. It is worth noting that L TA-PB@PAAm hydrogel had a significant bactericidal effect, and the bacterial survival rate was only 0.3%. Prussian blue has a wide range of functions, including antibacterial, antioxidant, enzyme-mimetic, and detoxifying properties. Among them, because of its good biocompatibility, biological stability, and excellent photothermal effect, it has been widely studied by scholars (Busquets and Estelrich, 2020). Tannic acid is a kind of natural polyphenol, which has antibacterial and antioxidant ability25. This antioxidant molecule can trap large amounts of free radicals and thus play a key role in wound healing. Because it is non-toxic and non-carcinogenic, it is used by many scholars to study wound repair. On the one hand, in this experiment, in addition to using heat to directly kill bacteria, we can also use near-infrared radiation to trigger the release of local high concentrations of tannic acid from the hydrogel, so as to synergistically enhance the antibacterial activity. Tannic acid, on the other hand, is a polyphenolic substance that is effective against bacteria while reducing antibiotic overuse resulting in irreversible resistance. Here we speculate that the main reason why the photothermal effect can effectively kill bacteria is that it inhibitions the enzyme activity of bacteria, because the optimal activity temperature of enzymes in bacteria is between 30°C and 40°C, however, the enzyme activity is strongly inhibited at a higher temperature12.

Meanwhile, the survival rate of *E. coli* was as follows (Figures 5A,E), NL control, L control, NL PB@PAAm, L PB@PAAm, NL TA@PAAm, L TA@PAAm, NL TA-PB@PAAm, and L TA-PB@PAAm was 100, 95.6, 96.2, 41.9, 99.4, 93.7, 94.8, and 42.8%, respectively. We found that compared with the control group, there was no difference in the number of bacterial colonies when *E. coli* was inoculated on the surface of L Control, NL PB@PAAm, N TA@PAAm, L TA@PAAm, or NL TA-PB@PAAm. When inoculated on the surface of L PB@PAAm and L TA-PB@PAAm, the number of bacterial colonies decreased significantly, which was about 40% of the control group. It has been reported in the literature that tannic acid is not effective in killing *E. coli*25, which may be related to its extensive drug resistance. Fortunately, the main bacteria involved in skin infections is *S. aureus*, and the photothermal effect can achieve a certain antibacterial function, to fill this defect.

The killing effect of photothermal hydrogel composites on *S. aureus* and *E. coli* was characterized by SEM (Figure 5B). It was observed that with the addition of TA or photothermal effects, the membranes of *S. aureus* and *E. coli* were shrunk. In the bacteria with both TA and Prussian blue photohydrothermal action, obvious destruction of the membrane was observed. The significant efficacy of our photothermal hydrogel composites was illustrated.

We further tested the sustained antibacterial of *S. aureus* and *E. coli* with composite photothermal hydrogels by the bacterial inhibition ring test for 48 h (Figures 5C,F, Supplementary Figure S5). In NL control and L PB@PAAm groups, the diameter of the inhibition ring was 0 cm. The diameters of the inhibition rings of NL TA@PAAm, L TA@PAAm, NL TA, NL TA-PB@PAAm, and L TA-PB@PAAm were 1.13, 1.27, 1.37, 1.63, and 1.77 cm, respectively. Compared with the control group, the results of NL TA@PAAm, L TA@PAAm, NL TA, NL TA-PB@PAAm, and L TA-PB@PAAm were statistically different. Interestingly, the thermal effect could not be effectively performed in the L PB@PAAm group perhaps due to the low water content in the plate. Since we can see that the surface of the hydrogel and the areas where it touched are free of bacteria, therefore, we can speculate that the photothermal effect of Prussian blue also has a certain bactericidal function in environments of a certain humidity. In summary, the above results indicated that illumination promoted the release of drugs, played a synergistic antibacterial effect and had the ability to produce a sustained release.

### *In Vivo* Biological Assessment

#### Wound Healing Evaluation

**FIGURE 6:**
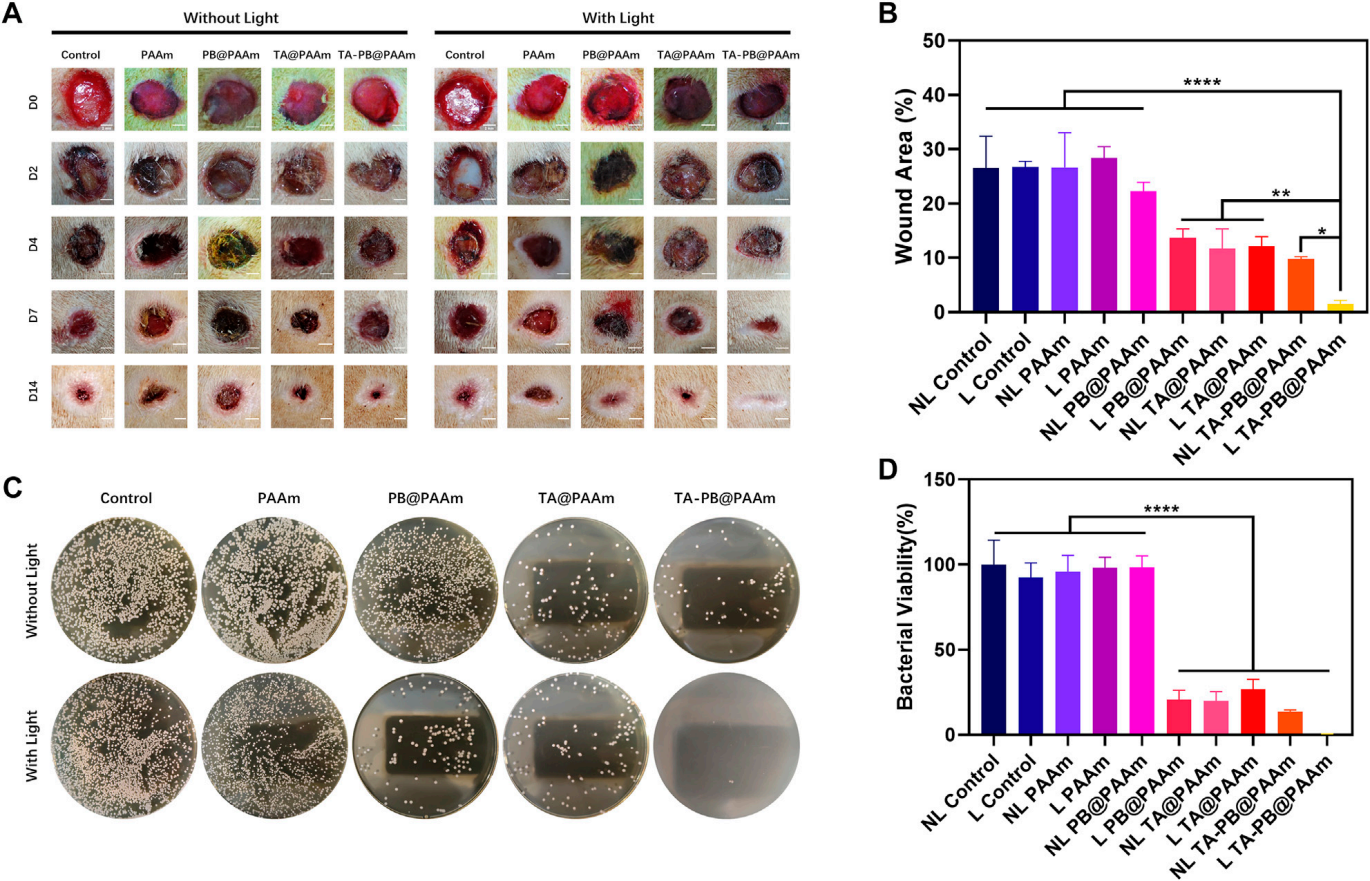
Wound healing in SD rats. **(A)** Results of skin repair of infectious skin defect in animal model. Scale bar = 2 mm. **(B)** Quantitative results of the areas of wound healing. **(C)** *In-vivo* bacteriostatic experiments using different hydrogels on *Staphylococcus aureus*. **(D)** Quantitative results of the areas with a survival rate of *Staphylococcus aureus*. Data are mean ± SD (n = 3, *p < 0.05,**p < 0.01, ****p < 0.0001).

The skin defects in all groups gradually healed (Figure 6A). From day 2 to day 4 after surgery, signs of skin infection were observed in the NL control, L control, NL PAAm, L PAAm, and NL PB@PAAm groups, while no signs of skin infection were observed in the other groups. By day 7, signs of infection were reduced in the NL Control, L Control, NL PAAm, L PAAm, and NL PB@PAAm groups. On day 14, infection in each group was significantly controlled, and skin healing in L PB@PAAm, NL TA@PAAm, L TA@PAAm, NL TA-PB@PAAm, and L TA-PB@PAAm was significantly higher than that in the other groups. Particularly, the skin healing in L TA-PB@PAAm was the best. The wound healing rate on day 14 was calculated **(** Figure 6B **)**. NL control, L control, NL PAAm, L PAAm, NL PB@PAAm, L PB@PAAm, NL TA@PAAm, L TA@PAAm, NL TA-PB@PAAm, and L TA-PB@PAAm decreased to 26.5, 26.7, 26.7, 28.4, 22.2, 13.7, 11.7, 12.1, 9.8, and 1.5%, respectively.

#### *In vivo* Bacterial Plate Counting Test

We counted the bacteria numbers in the skin defect wound of rats 24 h after surgery, and the survival rate of *S. aureus* was as follows **(** Figures 6C,D **)**. The survival rate in NL control, L control, NL PAAm, L PAAm, NL PB@PAAm, L PB@ PAAm, NL TA@ PAAm, L TA@ PAAm, NL TA-PB@ PAAm, and L TA-PB@ PAAm was 100, 92.4, 95.6, 98.0, 98.3, 20.6, 19.9, 26.7, 13.5, and 0.3%, respectively. The results were consistent with those of skin healing.

#### Histological Staining Evaluation

**FIGURE 7:**
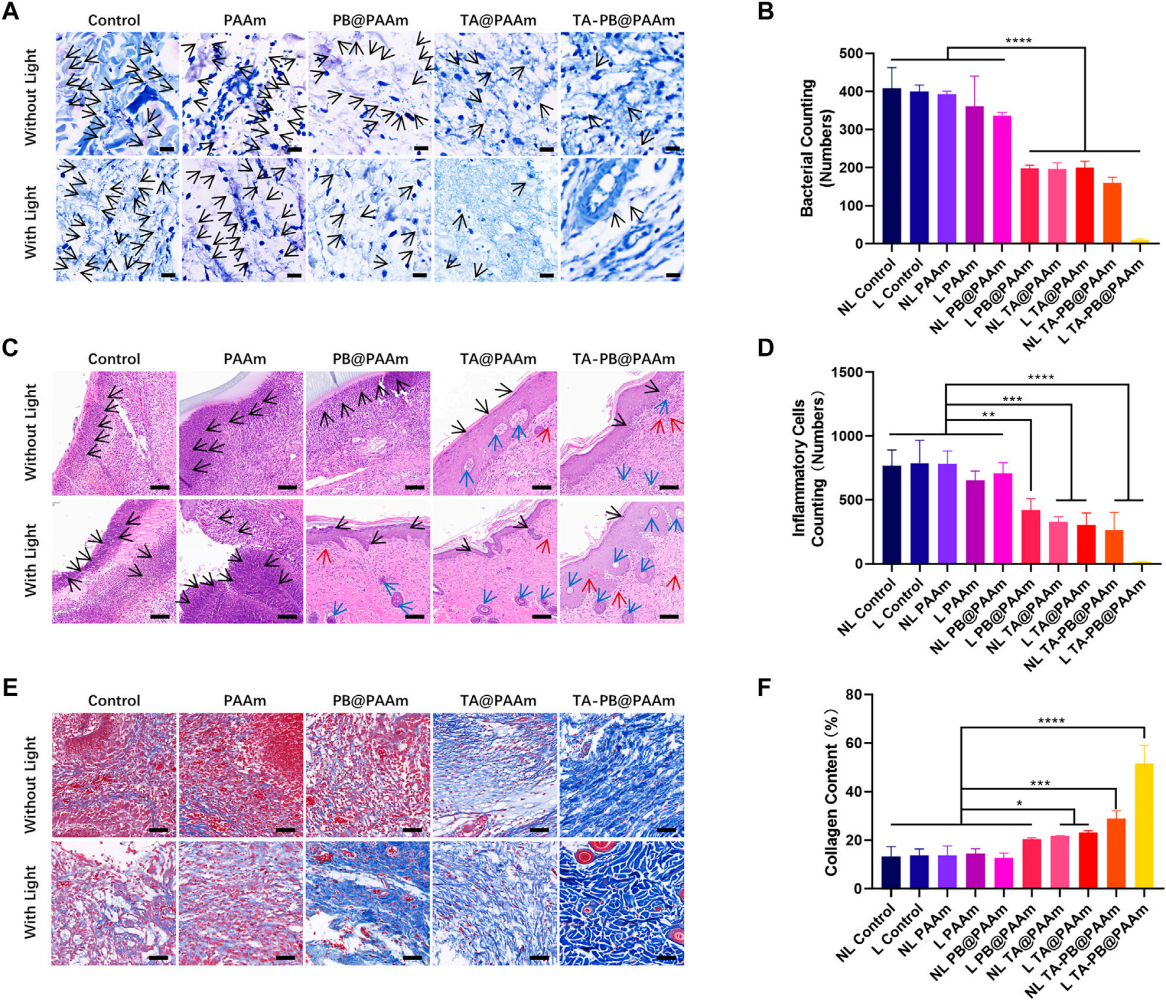
Histological staining evaluation. **(A)** Giemsa staining of infective skin defects shows the smallest number of bacteria for the L TA-PB@PAAm group. Black arrow represents bacteria. Scale bar = 20 μm. **(B)** Quantitative results of bacterial viability in Giemsa staining. **(C)** H and E staining showed skin repair results, neutrophils are fewer in the L PB@PAAm, N TA@PAAm, L TA@PAAm, NL TA-PB@PAAm, L TA-PB@PAAm groups. Black arrows represent inflammatory cells, red arrows represent newly formed vessels, and blue arrows represent newly formed hair follicles. Scale bar = 100 μm. **(D)** Quantitative results of inflammatory cells in H and E staining. **(E)** Masson’s trichrome staining shows the content of collagen are higher in the N TA@PAAm, L TA@PAAm, NL TA-PB@PAAm, L TA-PB@PAAm groups. **(F)** Quantitative results of collagen content in Masson’s trichrome staining. Data are mean ± SD (n = 3, *p < 0.05,**p < 0.01, ****p < 0.0001). Scale bar = 100 μm.

Histological analyses were also performed to evaluate the antimicrobial and tissue-repair properties of different composite photothermal hydrogels. In order to evaluate the antibacterial properties of different composite photothermal hydrogels, we used Giemsa staining to observe the number of bacteria (Figures 7A,B). We could see at day 2, a large number of bacteria appeared in the skin tissue of NL control, L control, NL PAAm, L PAAm, NL PB@ PAAm group, while L PB@ PAAm, NL TA@ PAAm, L TA@ PAAm, NL TA-PB@ PAAm, and L TA-PB@ PAAm group had relatively few bacteria in the skin tissue, and the bacteria content of the L TA-PB@PAM group was significantly lower than that of the other groups. We carried out quantitative counting of bacteria by Giemsa staining in different groups. The bacterial counting in NL control, L control, NL PAAm, L PAAm, NL PB@PAAm, L PB@PAAm, NL TA@PAAm, L TA@PAAm, NL TA-PB@PAAm, and L TA-PB@PAAm was 408.0 ± 54.6, 399.7 ± 17.2, 391.7 ± 9.0, 360.3 ± 80.1, 336.3 ± 8.6, 197.3 ± 9.0, 196.0 ± 16.5, 199.7 ± 17.1, 160.0 ± 14.9, and 10.0 ± 2.6, respectively.

In order to evaluate the tissue repair function of different composite photothermal hydrogels, we used H and E staining to observe the number of inflammatory cells and skin repair conditions (Figures 7C,D). On 14 days after surgery, in the NL Control, L Control, NL PAAm, L PAAm, and NL PB@PAAm groups, there was still necrotic tissue and a large number of neutrophils in the skin tissue, and poor tissue repair was also observed. However, the neutrophils in the L PB@PAAm, NL TA@PAAm, L TA@PAAm, NL TA-PB@PAAm, and L TA-PB@PAAm groups were relatively less, while vessels and hair follicles were observed. Compared with the other groups, the bacterial content of neutrophils in the L TA-PB@PAM group was significantly reduced, and the skin tissue was repaired well, and a large number of hair follicles and vessels were found. We carried out quantitative counting of bacteria by H and E staining in different groups. The number of inflammatory cells in NL control, L control, NL PAAm, L PAAm, NL PB@PAAm, L PB@ PAAm, NL TA@ PAAm, L TA@ PAAm, NL TA-PB@ PAAm, and L TA-PB@ PAAm was 766.0 ± 124.4, 786.7 ± 180.0, 781.3 ± 102.1, 654.7 ± 70.5, 709.3 ± 82.6, 418.7 ± 90.3, 328.7 ± 39.1, 304.3 ± 93.8, 264.7 ± 137.7, and 15.7 ± 2.5, respectively.

We also used Masson’s trichrome staining to observe the type I collagen and skin repair conditions (Figures 7E,F). Fourteen days after surgery, in the NL Control, L Control, NL PAAm, L PAAm, and NL PB@PAAm group, there were still muscle fibers and a large number of red blood cells in the skin tissue, and poor tissue repair was also observed. However, the type I collagen in the L PB@PAAm, NL TA@PAAm, L TA@PAAm, NL TA-PB@PAAm, and L TA-PB@PAAm groups were relatively higher. Compared with the other groups, the type I collagen in the L TA-PB@PAM group was significantly highest, and the skin tissue was repaired well. We carried out quantitative counting of type I collagen in different groups. The content of type I collagen in NL control, L control, NL PAAm, L PAAm, NL PB@PAAm, L PB@ PAAm, NL TA@ PAAm, L TA@ PAAm, NL TA-PB@ PAAm, and L TA-PB@ PAAm was 13.4, 13.7, 13.8, 14.5, 12.7, 20.6, 21.8, 23.1, 28.9, and 51.6%, respectively.

The main reasons for this result are as follows: 1) Photothermal effect synergistic antibacterial can effectively kill bacteria and reduce tissue necrosis and cell destruction caused by toxins produced by bacteria, such as *α*-toxin produced by *S. aureus*, which is a kind of membrane penetrating toxin and can cause cell death. 2) Bacterial toxins can weaken the proliferation and differentiation of fibroblasts and reduce the ability of skin repair, while the antibacterial effect of composite photothermal hydrogels can indirectly enhance the repair ability of fibroblasts. 3) TA and PB have anti-inflammatory and anti-oxidant effects, and the good cellular compatibility of the composite photothermal hydrogel provides a stable environment for the growth of fibroblasts and further promotes tissue healing. 4) Thermal-disrupting interface induced mitigation (TRIM) provided by hydrogel can reduce the thermal effect and effectively protect normal tissues, so that the photothermal temperature reaches below 50°C and cells can be effectively protected from high-temperature damage. Therefore, combined with the above advantages, L TA-PB@PAAm hydrogel can better resist bacterial and repair tissue.

#### Histocompatibility Evaluation

**FIGURE 8:**
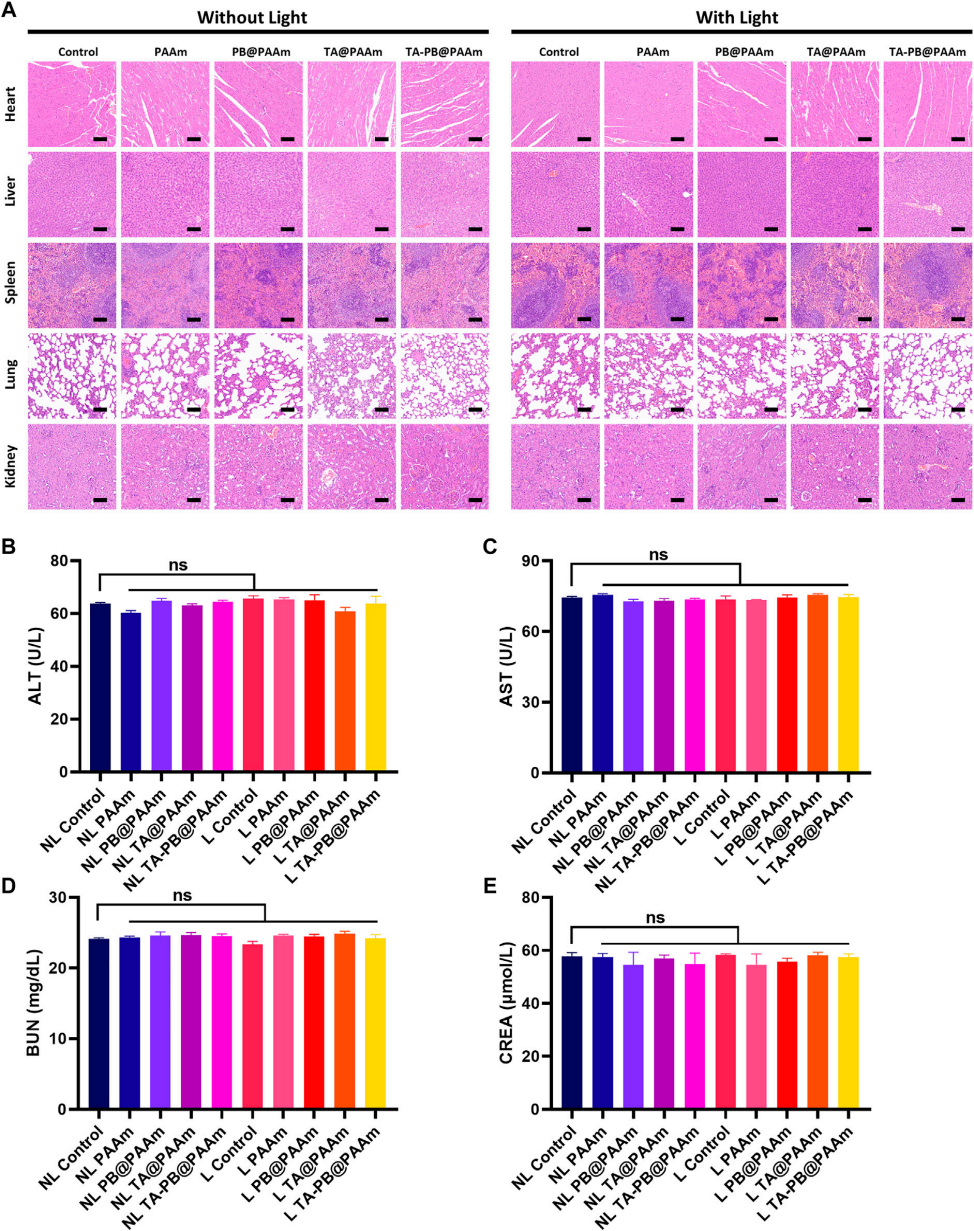
a H and E staining of heart, liver, spleen, lung and kidney. Scale bar = 100 μm. b Results of ALT. c Results of AST. d Results of BUN. e Results of CREA. Data are mean ± SD (n = 3, ns = no statistical difference).

H and E staining was performed on the sections of vital organs to further evaluate the biocompatibility of different samples (Figure 8A). Compared with the control group, there were no obvious organ damages or inflammatory lesions in the heart, liver, spleen, lung, and kidney of the composite photothermal hydrogel group. In addition, the results of liver and renal function were tested. ALT, AST, BUN, and CREA had no significant difference among different photothermal hydrogels (Figures 8B–E). These results showed that the composite photothermal hydrogels had good histocompatibility.

## Conclusions

In conclusion, we fabricated a novel composite photothermal hydrogel with a simple synthetic method, triggering drug release rate, thermal-disrupting interface induced mitigation alleviating heat damage, transparent morphology observing wound healing, mechanical stability, strong adhesion, high swelling rate absorbing exudate, good biocompatibility, synergistic antibacterial ability, and excellent promotion of skin repair ability. As a new type of composite biomaterial, it has a strong guiding significance for the treatment of infectious skin defects in clinical use.

## Experimental Section

### Materials

Acrylamide, ferric chloride, potassium ferricyanide, citric acid, tannic acid, ammonium persulfate (APS), and 4-sulfanylbutanimidamide (MBA) were purchased from Sinopharm Chemical Reagent Co., Ltd. Solvents and buffer solutions were purchased from Servicebio (China). Lysogeny broth agar plates (LB), Fetal bovine serum, D-MEM medium were purchased from Thermo Fisher Scientific (China). Penicillin–streptomycin solution (PS) was purchased from HyClone (China). CCK-8 kits were purchased from Yeasen Biotech Co., Ltd. (China). NIH-3T3 cells (CL-0171) were purchased from Procell Life Science and Technology Co., Ltd. *S. aureus* and *E. coli* were purchased from China Center of Industrial Culture Collection (CICC, Beijing, China). All agents were used as received and used without further purification.

### Synthesis of Materials

#### Fabrication of PBNPs

The synthesis of Prussian blue nanoparticles is based on previously reported methods (Maaoui et al., 2016). The brief description is as follows. Added 0.5 mmol (12.5% W/V %) citric acid to 20 ml ferric chloride solution containing 0.02 mmol (0.16% W/V %) and stirred it evenly at 60 °C to prepare 1 mM ferric chloride solution A. Then, 0.5 mmol (12.5% W/V %) citric acid was added to 20 ml of 0.02 mmol (0.33% W/V %) potassium ferricyanide solution to prepare 1 mM solution B. Dropped solution B dropwise into solution A (80–100 drops per minute) at 60 °C and stirred evenly. Finally, Prussian blue nanoparticles with a concentration of 0.5 mM were prepared after a 12 h reaction. The obtained product was purified with a dialysis membrane (MW: 1.4 × 10^3^ Da) for 48 h to remove the excess reactants.

#### Fabrication of PAAm and PB@PAAm

The preparation method of polyacrylamide hydrogels is based on previous reports (Ning et al., 2014). The brief description is as follows: 1.42 g (0.02 mol) of acrylamide (142% W/V%), 4-sulfanylbutanimidamide (MBA) 3 mg (0.3% W/V%), then added 10 ml DI water to mix AAm and MBA solution, or added the appropriate amount of PB and stirred equally. Next step, added ammonium persulfate (APS) 4.7 mg (0.47% W/V %) to the above solution and stirred evenly. Polyacrylamide hydrogel or polyacrylamide hydrogel containing PB (abbreviated as PAAm and PB@PAAm) was prepared by placing the above solution in a 45°C water bath for 3 h. The obtained product was purified with a dialysis membrane for 48 h to remove the excess reactants.

#### Fabrication of TA@PAAm and TA-PB@PAAm by Immersion Method

Tannic acid is loaded by immersion method as previously reported (Zheng et al., 2018). The detailed steps are as follows: Tannic acid powder of different qualities was weighed and prepared into TA solutions of different concentrations (0.5, 1, 5 mg/ml). The prepared PAAm and PB@PAAm were immersed in the solution overnight, and the prepared hydrogel was centrifuged to remove the unattached TA. The obtained hydrogel was then washed three times with DI water. Finally, TA@PAAm and TA-PB@PAAm were prepared for further characterization.

### Physical Characterization of Composite Photothermal Hydrogel

Observed the morphological appearance of TA-PB@PAAm. TA-PB@PAAm hydrogel was first frozen using liquid nitrogen, and then freeze-dried. After that, a scanning electron microscope (SEM, Quanta-200, United States) was used to observe the structure and surface morphology of TA-PB@PAAm and PB nanoparticles at the microscopic level. At the same time, FTIR and XRD were used to characterize the success of the material preparation.

The characteristic ultraviolet wavelength (UV) absorption at 700 nm of PB was used to quantify the content of PB and UV absorption at 270 nm of TA was used to quantify the content of TA in PB@PAAm and TA-PB@PAAm, which was measured by a spectrophotometer (UV- 2,450, Shimadzu, Japan). Measured the weight of drugs loaded on PAAm hydrogel (Wd), and the weight of PAAm hydrogel (Wg). The drugs loading ratio was calculated according to the following Eq. 1 (Vieira et al., 2019):

Drugs loading ratio(%)= Wd/(Wd +Wg)×100% (1)

Besides, Zeta potentials of PB, TA, TA + PB, andPAAm hydrogel precursor solution and TA-PB@PAAm hydrogel precursor solution were measured with a zeta-sizer instrument (Nano ZS, Malvern Instruments, United Kingdom).

The swelling ratio was calculated by following the conventional gravimetric method. Hydrogels were lyophilized and the dry weight of the hydrogels was recorded (Wi). Hydrogels were immersed in PBS (pH 7.4, 37°C) and measured the rehydrated weight (Wr) after specific time points. The swelling ratio was determined by the Eq. 2 (Kumar et al., 2017).

Swelling ratio(%)= (Wr−Wi)/Wi ×100% (2)

The viscoelastic properties of TA-PB@PAAm were characterized by dynamic shear rotation measurement on Anton Paar MCR302 rheometer. Under the constant strain mode, the mechanical spectrum was recorded in the frequency range of 0.01–1 rad/s (Hz).

### Photothermal Property

PB@PAAm or TA-PB@PAAm hydrogels (0.5 g) were formed in a 2 ml Eppendorf tube and then irradiated under fiber-coupled continuous semiconductor diode laser (808 nm, Beijing Viasho Technology Co., Ltd. China) with different PB concentrations (0, 0.1, 0.2, 0.3, 0.4 mg/ml) or a laser density of 0.25, 0.5, 0.75, 1.0W/cm^2^ for 5 min. Then the temperature was recorded using an infrared thermal camera (Flir C2, United States) and data were recorded every 30 s.

### *In vitro* NIR Light-Triggered Drug Release

Release of TA from TA-PB@PAAm hydrogels (2.5% drug loading) was determined in DI water. 4ml of DI water was placed in a 5 ml Eppendorf tube containing 1 g of TA-PB@PAAm hydrogels. TA-PB@PAAm hydrogels were irradiated using a fiber-coupled continuous semiconductor diode laser (808 nm, Beijing Viasho Technology Co., Ltd. China) with a power of 0.5W/cm^2^ for 10 min, then followed by an interval of 30 min and the procedure was repeated three times. Before switching treatment conditions, 1 ml solution was taken and 1 ml PBS was added to keep the total amount of solution at 4 ml. Finally, a spectrophotometer (UV-2450, Shimadzu, Japan) was used to analyze the above solution to determine the release amount of TA. In comparison, the hydrogel without NIR light irradiation was set as a control.

### Cell Compatibility of NIH-3T3 With TA-PB@PAAm

To culture NIH-3T3 cells, we used Complete Dulbecco’s modified Eagle’s medium, which contained 10% fetal bovine serum and 2% penicillin and streptomycin. Then put NIH-3T3 cells in an incubator at 37°C under 5% CO2.

For the CCK-8 test, the composite photothermal hydrogel was co-cultured with cells using a 48-well plate. NIH-3T3 cells with the number of 2*104 were inoculated into the blank well as the control group. The experimental group was inoculated on the top of PAAm, PB@PAAm, and TA-PB@PAAm, respectively. Another four groups of cells incubated on different kinds of hydrogel were treated with 808 nm NIR light. The medium should be changed every 24 h after washing 3 times with PBS. At the time point at 1, 3, and 5 days, CCK-8 medium and D-MEM medium were diluted at a ratio of 1:10 and incubated in each well for 2 h. Then, 100 μl of supernatant was taken and transferred to a 96-well plate. A microplate reader (Infinite M200Pro, Tecan, Switzerland) was used to detect the absorbance value at 450 nm wavelength (Xu et al., 2015). The cell metabolic rate was calculated according to the OD value. The experiment was repeated three times for each group of three samples.

### *In Vitro* Bactericidal Test of Composite Photothermal Hydrogel

Gram-positive bacteria *S. aureus* and Gram-negative bacteria *E. coli* were cultured separately in an incubator at 37°C with fresh lysogeny broth (LB) medium (Thermo Fisher Scientific, China), which contained 5 mg/ml yeast extract, 10 mg/ml tryptone, and 10 mg/ml NaCl.

Selected overnight cultured *S. aureus* were diluted 10 times in LB medium and were diluted to 1 × 108 CFU/ml after 2 h of culture. Then take 2 ml of *S. aureus* suspension into a 2.5 ml Eppendorf tube containing 1 ml of TA-PB@PAAm hydrogel. After being irradiated with 808 nm (0.5W/cm2) near-infrared laser for 10 min, 100 μl diluted bacterial suspension (1:105 dilution) was taken and coated onto a TSA plate. After incubation for 24 h, the number of culture colonies was counted. Seven other groups (NL control, L control, NL PB@PAAm, L PB@PAAm, NL TA@PAAm, L TA@PAAm, and NL TA-PB@PAAm) were set to compare the antibacterial effect (NL for without NIR light, L for with NIR light). Three parallel samples were set in each group. The antibacterial activity of PB@PAAm hydrogels toward *E. coli in vitro* was tested in a similar way.

In order to observe the killing effect of photothermal hydrogel composites on *S. aureus* and *E. coli*, scanning electronic microscopic (SEM) characterization was carried out. The grouping is (NL Control, L PAAm, L PB@PAAm, NL TA@PAAm, and L TA-PB@PAAm).

In order to study the continuous antibacterial ability of the material, the antibacterial activity of PB@PAAm hydrogels toward *S. aureus* and *E. Coli* was tested by the bacterial inhibition ring test. Specifically, 100 μl of *S. aureus* suspension with the concentration of 1*106 CFU/ml was plated onto the TSA plate. Then the L TA-PB@PAAm was transferred to the *S. aureus*-coated TSA plate, irradiated with 808 nm (0.5W/cm^2^) near-infrared laser for 10 min. Six other groups (NL control, L PB@PAAm, NL TA, NL TA@PAAm, L TA@PAAm, and NL TA-PB@PAAm) were set to compare the antibacterial activity. NL control group used sterile filter paper. NL TA group used sterile filter paper soaked in TA solution and dried. They were placed on the surface of the plate. NL TA@PAAm, NL TA-PB@PAAm were put on the surface of the plate. The above materials were not given light treatment. L PB@PAAm and L TA@PAAm were given light for 10 min. Three parallel samples were set in each group. After incubation at 37°C for 48 h, the diameters of the inhibition area were determined and the antibacterial activities were compared. The procedures are the same for the bacterial inhibition ring test of *E. coli*.

### *In Vivo* Biological Assessment

#### Infective Skin Defect Model in SD Rats

Forty 8-week-old male SD rats, weighing 250–300 g, were provided by the Department of Animal Science and Ethics Committee, Xiangya Hospital, Central South University. The treatment of animals in the experiment conforms to the requirements of animal ethics according to the guidelines, principles, and procedures for the care and use of laboratory animals. To establish the *S. aureus*-infected skin defect model in rat, 100 μl (1 × 108 CFU/ml) of *S. aureus* in PBS was injected subcutaneously into the back of the rats. After infection for 2 days, the rats were randomly divided into 10 groups (n = 4). The manufacturing method of the skin defects is reported in the literature1. The detailed method was that after 12 h of fasting, the rats were anesthetized with 2% pentobarbital sodium and skinned. Full-thickness skin defects were applied with an 8 mm diameter circular tissue extractor and then treated in different groups (NL control, L control, NL PAAm, L PAAm, NL PB@PAAm, L PB@ PAAm, NL TA@ PAAm, L TA@ PAAm, NL TA-PB@ PAAm, and L TA-PB@ PAAm), respectively. Vital signs of the rats were determined after operation.

#### in vivo Bacterial Plate Counting Test

After feeding for 24 h, the treated rats were photographed and killed, and the infected tissues were collected and homogenized with a homogenizer (500 μl normal saline). The homogenized supernatant was diluted at different multiples (10, 100, 1,000, 10,000) and inoculated on the TSA plat. After overnight incubation, colonies were counted to evaluate the *in vivo* antimicrobial activity.

#### Wound Healing and Histological Staining Evaluation

After feeding for 24 h, a group of rats was sacrificed by overinjection of pentobarbital sodium. Skin tissues were taken and soaked in 10% formalin solution for 24 h, and then embedded in paraffin for sectioning. The tissue sections were stained with Giemsa and captured using the Optical Microscope (Leica DMIL LED, Germany). After 14 days of feeding, all rats were sacrificed by overinjection of pentobarbital sodium. The skin defect was photographed, and defect area was calculated and the repair ability of the skin defect was evaluated. Then, skin tissues were taken and immersed in 10% formalin solution for 24 h, and then embedded in paraffin wax for sectioning. Hematoxylin and eosin (H and E) staining and Masson staining were performed and then the images captured.

#### Histocompatibility Evaluation

The tissues of the heart, liver, spleen, lung, and kidney were obtained and prepared into paraffin sections and performed with H and E staining. In addition, the liver and renal functions of rats in different groups were also evaluated, including ALT, AST, BUN, and CREA.

### Statistical Analysis

Data analysis was performed using the GraphPad Instat 3.0 program (GraphPad Software, La Jolla, CA). The results were expressed as mean ± standard deviation. Student’s t-test was used to analyze the comparison between two groups. A one-way ANOVA test was used to analyze the comparison between three or more groups. The q test (Student–Newman–Keuls method) was used for multiple comparisons between the groups when *p* < 0.05 was detected in the one-way ANOVA test. Among all results, *p* < 0.05 was statistically significant.

## Associated Content

### Supporting Information

FTIR characterization of different components (Supplementary Figure S1); XRD characterization of different components (Supplementary Figure S2); the standard curve of tannic acid in different concentrations, absorbance profiles of tannic acid in different concentrations (Supplementary Figure S3); absorbance values of NIH-3T3 cells after intervention with different concentrations of tannic acid or Prussian blue (Supplementary Figure S4); bacterial inhibition ring test of *E. coli* (Supplementary Figure S5).

## Data Availability

The raw data supporting the conclusions of this article will be made available by the authors, without undue reservation.

## References

[B1] Borzenkov M. D’Alfonso L. Polissi A. Sperandeo P. Collini M. Dacarro G. (2019). Novel Photo-Thermally Active Polyvinyl Alcohol-Prussian Blue Nanoparticles Hydrogel Films Capable of Eradicating Bacteria and Mitigating Biofilms. Nanotechnology 30 (29), 295702. 10.1088/1361-6528/ab15f9 31025630

[B2] Busquets M. A. Estelrich J. (2020). Prussian Blue Nanoparticles: Synthesis, Surface Modification, and Biomedical Applications. Drug Discov. Today 25, 1431–1443. 10.1016/j.drudis.2020.05.014 32492486

[B3] Cai S. Qian J. Yang S. Kuang L. Hua D. (2019). Acetylcysteine-decorated Prussian Blue Nanoparticles for strong Photothermal Sterilization and Focal Infection Treatment. Colloids Surf. B: Biointerfaces 181, 31–38. 10.1016/j.colsurfb.2019.05.007 31121379

[B4] Cao C. Ge W. Yin J. Yang D. Wang W. Song X. (2020). Mesoporous Silica Supported Silver-Bismuth Nanoparticles as Photothermal Agents for Skin Infection Synergistic Antibacterial Therapy. Small 16 (24), e2000436. 10.1002/smll.202000436 32406205

[B5] Chen T. Chen Y. Rehman H. U. Chen Z. Yang Z. Wang M. (2018). Ultratough, Self-Healing, and Tissue-Adhesive Hydrogel for Wound Dressing. ACS Appl. Mater. Inter. 10 (39), 33523–33531. 10.1021/acsami.8b10064 30204399

[B6] Dai X. Zhao Y. Yu Y. Chen X. Wei X. Zhang X. (2017). Single Continuous Near-Infrared Laser-Triggered Photodynamic and Photothermal Ablation of Antibiotic-Resistant Bacteria Using Effective Targeted Copper Sulfide Nanoclusters. ACS Appl. Mater. Inter. 9 (36), 30470–30479. 10.1021/acsami.7b09638 28832120

[B7] Fang J. Li P. Lu X. Fang L. Lü X. Ren F. (2019). A strong, Tough, and Osteoconductive Hydroxyapatite Mineralized Polyacrylamide/dextran Hydrogel for Bone Tissue Regeneration. Acta Biomater. 88, 503–513. 10.1016/j.actbio.2019.02.019 30772515

[B8] Gan D. Han L. Wang M. Xing W. Xu T. Zhang H. (2018). Conductive and Tough Hydrogels Based on Biopolymer Molecular Templates for Controlling *In Situ* Formation of Polypyrrole Nanorods. ACS Appl. Mater. Inter. 10 (42), 36218–36228. 10.1021/acsami.8b10280 30251533

[B9] Han D. Li Y. Liu X. Li B. Han Y. Zheng Y. (2020). Rapid Bacteria Trapping and Killing of Metal-Organic Frameworks Strengthened Photo-Responsive Hydrogel for Rapid Tissue Repair of Bacterial Infected Wounds. Chem. Eng. J. 396, 1. 10.1016/j.cej.2020.125194

[B10] Hao S. Shao C. Meng L. Cui C. Xu F. Yang J. (2020). Tannic Acid-Silver Dual Catalysis Induced Rapid Polymerization of Conductive Hydrogel Sensors with Excellent Stretchability, Self-Adhesion, and Strain-Sensitivity Properties. ACS Appl. Mater. Inter. 12 (50), 56509–56521. 10.1021/acsami.0c18250 33270440

[B11] Hu B. Berkey C. Feliciano T. Chen X. Li Z. Chen C. (2020). Thermal-disrupting Interface Mitigates Intercellular Cohesion Loss for Accurate Topical Antibacterial Therapy. Adv. Mater. 32 (12), e1907030. 10.1002/adma.201907030 32072703 PMC7702719

[B12] Jiang T. He J. Sun L. Wang Y. Li Z. Wang Q. (2018). Highly Efficient Photothermal Sterilization of Water Mediated by Prussian Blue Nanocages. Environ. Sci. Nano 5 (5), 1161–1168. 10.1039/c7en01245d

[B13] Kumar M. Nandi S. K. Kaplan D. L. Mandal B. B. (2017). Localized Immunomodulatory Silk Macrocapsules for Islet-like Spheroid Formation and Sustained Insulin Production. ACS Biomater. Sci. Eng. 3 (10), 2443–2456. 10.1021/acsbiomaterials.7b00218 33445302

[B14] Li J. Liu X. Tan L. Cui Z. Yang X. Liang Y. (2019). Zinc-doped Prussian Blue Enhances Photothermal Clearance of *Staphylococcus aureus* and Promotes Tissue Repair in Infected Wounds. Nat. Commun. 10 (1), 4490. 10.1038/s41467-019-12429-6 31582736 PMC6776522

[B15] Li M. Liu X. Tan L. Cui Z. Yang X. Li Z. (2018). Noninvasive Rapid Bacteria-Killing and Acceleration of Wound Healing through Photothermal/photodynamic/copper Ion Synergistic Action of a Hybrid Hydrogel. Biomater. Sci. 6 (8), 2110–2121. 10.1039/c8bm00499d 29882566

[B16] Liu L. Ge C. Zhang Y. Ma W. Su X. Chen L. (2020). Tannic Acid-Modified Silver Nanoparticles for Enhancing Anti-biofilm Activities and Modulating Biofilm Formation. Biomater. Sci. 8 (17), 4852–4860. 10.1039/d0bm00648c 32734981

[B17] Luo Y. Li J. Liu X. Tan L. Cui Z. Feng X. (2019). Dual Metal-Organic Framework Heterointerface. ACS Cent. Sci. 5 (9), 1591–1601. 10.1021/acscentsci.9b00639 31572786 PMC6764158

[B18] Maaoui H. Jijie R. Pan G.-H. Drider D. Caly D. Bouckaert J. (2016). A 980 Nm Driven Photothermal Ablation of Virulent and Antibiotic Resistant Gram-Positive and Gram-Negative Bacteria Strains Using Prussian Blue Nanoparticles. J. Colloid Interf. Sci. 480, 63–68. 10.1016/j.jcis.2016.07.002 27405072

[B19] Mao C. Xiang Y. Liu X. Cui Z. Yang X. Li Z. (2018). Repeatable Photodynamic Therapy with Triggered Signaling Pathways of Fibroblast Cell Proliferation and Differentiation to Promote Bacteria-Accompanied Wound Healing. ACS Nano 12 (2), 1747–1759. 10.1021/acsnano.7b08500 29376340

[B20] Mukherjee S. Kotcherlakota R. Haque S. Das S. Nuthi S. Bhattacharya D. (2020). Silver Prussian Blue Analogue Nanoparticles: Rationally Designed Advanced Nanomedicine for Multifunctional Biomedical Applications. ACS Biomater. Sci. Eng. 6 (1), 690–704. 10.1021/acsbiomaterials.9b01693 33463227

[B21] Ngo-Duc T. T. Alibay Z. Plank J. M. Cheeney J. E. Haberer E. D. (2020). Gold-Decorated M13 I-Forms and S-Forms for Targeted Photothermal Lysis of Bacteria. ACS Appl. Mater. Inter. 12 (1), 126–134. 10.1021/acsami.9b15682 31800209

[B22] Ninan N. Forget A. Shastri V. P. Voelcker N. H. Blencowe A. (2016). Antibacterial and Anti-inflammatory pH-Responsive Tannic Acid-Carboxylated Agarose Composite Hydrogels for Wound Healing. ACS Appl. Mater. Inter. 8 (42), 28511–28521. 10.1021/acsami.6b10491 27704757

[B23] Ning C. Logsetty S. Ghughare S. Liu S. (2014). Effect of Hydrogel Grafting, Water and Surfactant Wetting on the Adherence of PET Wound Dressings. Burns 40 (6), 1164–1171. 10.1016/j.burns.2013.12.024 24485358

[B24] Sahiner N. Sagbas S. Sahiner M. Silan C. Aktas N. Turk M. (2016). Biocompatible and Biodegradable poly(Tannic Acid) Hydrogel with Antimicrobial and Antioxidant Properties. Int. J. Biol. Macromolecules 82, 150–159. 10.1016/j.ijbiomac.2015.10.057 26526171

[B25] Sharma S. Chakraborty N. Jha D. Gautam H. K. Roy I. (2020). Robust Dual Modality Antibacterial Action Using Silver-Prussian Blue Nanoscale Coordination Polymer. Mater. Sci. Eng. C 113, 110982. 10.1016/j.msec.2020.110982 32487399

[B26] Van Den Bulcke A. I. Bogdanov B. De Rooze N. Schacht E. H. Cornelissen M. Berghmans H. (2000). Structural and Rheological Properties of Methacrylamide Modified Gelatin Hydrogels. Biomacromolecules 1 (1), 31–38. 10.1021/bm990017d 11709840

[B27] Vieira S. da Silva Morais A. Garet E. Silva-Correia J. Reis R. L. González-Fernández Á. (2019). Self-mineralizing Ca-Enriched Methacrylated Gellan Gum Beads for Bone Tissue Engineering. Acta Biomater. 93, 74–85. 10.1016/j.actbio.2019.01.053 30708066

[B28] Wang Z. Yu B. Alamri H. Yarabarla S. Kim M.-H. Huang S. D. (2018). KCa(H2O)2[FeIII(CN)6]⋅H2O Nanoparticles as an Antimicrobial Agent againstStaphylococcus Aureus. Angew. Chem. Int. Ed. 57 (8), 2214–2218. 10.1002/anie.201713177 PMC6358163 29392801

[B29] Xu M. McCanna D. J. Sivak J. G. (2015). Use of the Viability Reagent PrestoBlue in Comparison with alamarBlue and MTT to Assess the Viability of Human Corneal Epithelial Cells. J. Pharmacol. Toxicol. Methods 71, 1–7. 10.1016/j.vascn.2014.11.003 25464019

[B30] Xue H. Hu L. Xiong Y. Zhu X. Wei C. Cao F. (2019). Quaternized Chitosan-Matrigel-Polyacrylamide Hydrogels as Wound Dressing for Wound Repair and Regeneration. Carbohydr. Polym. 226, 115302. 10.1016/j.carbpol.2019.115302 31582049

[B31] Yan H. Ni H. Jia J. Shan C. Zhang T. Gong Y. (2019). Smart All-In-One Thermometer-Heater Nanoprobe Based on Postsynthetical Functionalization of a Eu(III)-Metal-Organic Framework. Anal. Chem. 91 (8), 5225–5234. 10.1021/acs.analchem.8b05960 30905160

[B32] Yang Y. Zhu W. Dong Z. Chao Y. Xu L. Chen M. (2017). 1D Coordination Polymer Nanofibers for Low-Temperature Photothermal Therapy. Adv. Mater. 29 (40), 1. 10.1002/adma.201703588 28833643

[B33] Yu P. Han Y. Han D. Liu X. Liang Y. Li Z. (2020). *In-situ* Sulfuration of Cu-Based Metal-Organic Framework for Rapid Near-Infrared Light Sterilization. J. Hazard. Mater. 390, 122126. 10.1016/j.jhazmat.2020.122126 32006853

[B34] Zhang Y. Fu H. Liu D.-E. An J. Gao H. (2019). Construction of Biocompatible Bovine Serum Albumin Nanoparticles Composed of Nano Graphene Oxide and AIEgen for Dual-Mode Phototherapy Bacteriostatic and Bacterial Tracking. J. Nanobiotechnol 17 (1), 104. 10.1186/s12951-019-0523-x PMC6785860 31601275

[B35] Zheng Y. Liang Y. Zhang D. Sun X. Liang L. Li J. (2018). Gelatin-Based Hydrogels Blended with Gellan as an Injectable Wound Dressing. ACS Omega 3 (5), 4766–4775. 10.1021/acsomega.8b00308 30023901 PMC6044880

[B36] Zhou D. Gao Y. A S. Xu Q. Meng Z. Greiser U. (2016). Anticancer Drug Disulfiram for *In Situ* RAFT Polymerization: Controlled Polymerization, Multifacet Self-Assembly, and Efficient Drug Delivery. ACS Macro Lett. 5 (11), 1266–1272. 10.1021/acsmacrolett.6b00777 35614738

