# Supplementary material for: Triggering Drug Release and Thermal-Disrupting Interface Induced Mitigation of Composite Photothermal Hydrogel Treating Infectious Wounds
Source: Front Bioeng Biotechnol. 2021 Dec 13;9:796602. doi: 10.3389/fbioe.2021.796602 (PMC8710761; doi:10.3389/fbioe.2021.796602)
Supplement: Supplementary file 1 [file DataSheet1.PDF]

# Triggering drug release and thermal-disrupting interface induced mitigation of composite photothermal hydrogel treating infectious wounds

**Long Hua[1, 2, 3], Hu Qian[1], Ting Lei[1], Wenbin Liu[1], Xi He[1], Pengfei Lei[1, 2#]* and Yihe Hu[1, 2#]***

**1** Department of Orthopedics, Xiangya Hospital Central South University, Hunan Engineering Research Center of Biomedical Metal and Ceramic Implants, Changsha, Hunan, P. R. China;

**2** Department of Orthopedics, The First Affiliated Hospital, Medical College of Zhejiang University, Hangzhou, P. R. China;

**3** The Sixth Affiliated Hospital, Xinjiang Medical University, Urumqi, P. R. China.

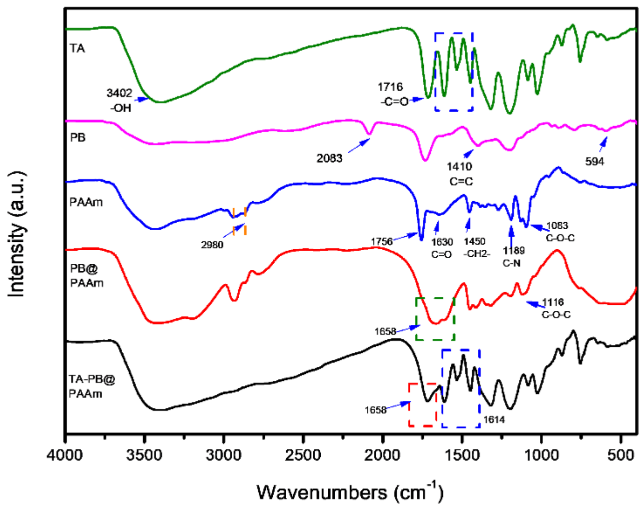


**Figure S1.** FTIR characterization of different components.

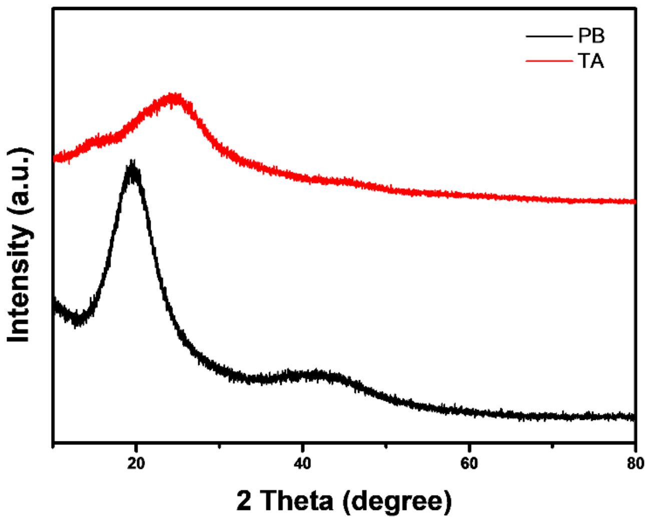


**Figure S2.** XRD characterization of different components.

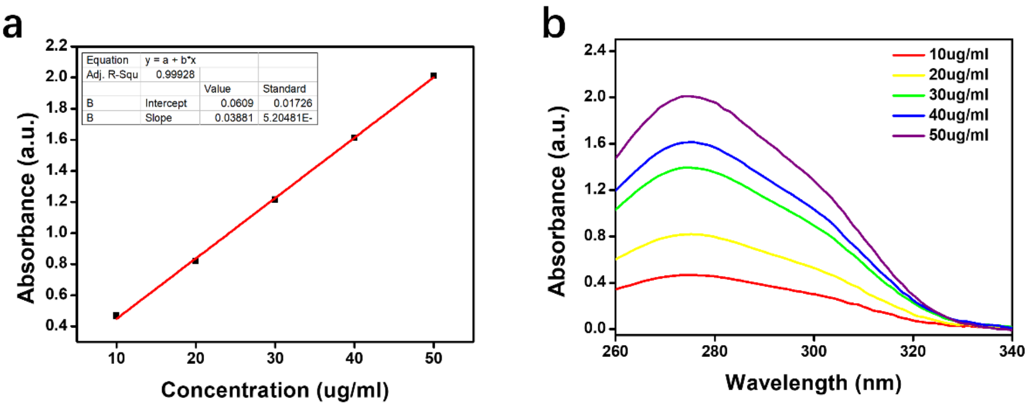


**Figure S3.** Results of tannic acid in UV-spectrum experiment. (a) Standard curve of tannic acid in different concentrations. (b) Absorbance profiles of tannic acid in different concentrations.

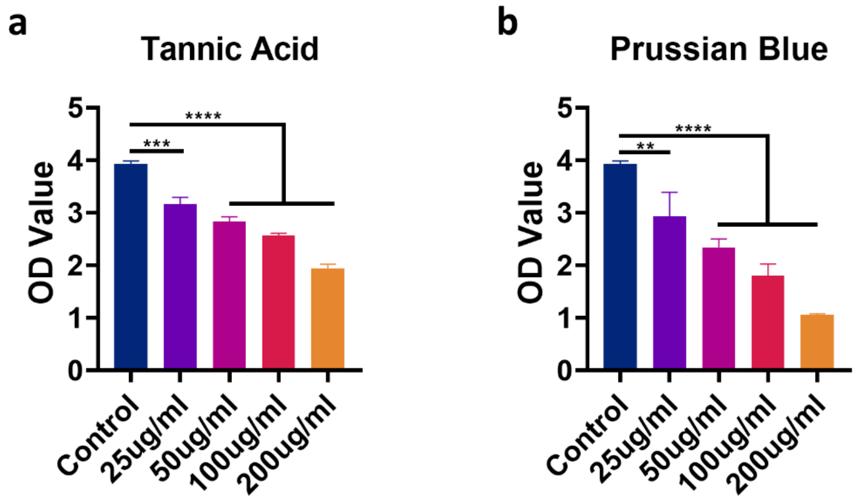


**Figure S4.** Determination of the 50% lethal dose of tannic acid and Prussian blue by CCK8 experiment. (a) Absorbance values of NIH-3T3 cells after intervention with different concentrations of tannic acid. (b) Absorbance values of different concentrations of Prussian blue after intervention in NIH-3T3 cells. Data are mean ± SD (n = 3,** $p < 0.01$, *** $p < 0.001$, **** $p < 0.0001$).

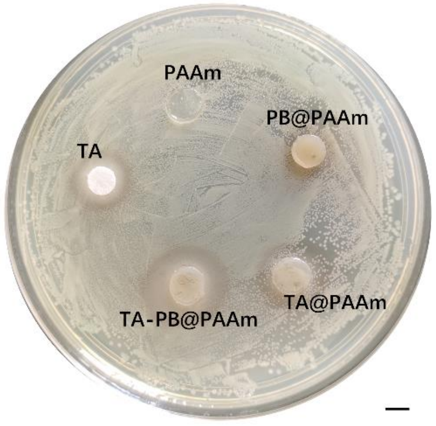


**Figure S5.** Bacterial inhibition ring test of *Escherichia coli*. There was no obvious bacterial inhibition ring around the hydrogel. Scale bar = 5 mm.
