# Supplementary material for: Triggering Drug Release and Thermal-Disrupting Interface Induced Mitigation of Composite Photothermal Hydrogel Treating Infectious Wounds
Source: Front Bioeng Biotechnol. 2021 Dec 13;9:796602. doi: 10.3389/fbioe.2021.796602 (PMC8710761; doi:10.3389/fbioe.2021.796602)
Supplement: Supplementary file 2 [file DataSheet2.docx]

**Figure 1.** Schematic diagram of Prussian blue nanoparticles and tannic acid drug particles loaded on acrylamide hydrogel for the elimination of bacteria and promotion of skin repair through the synergistic photothermal and antibacterial action of the drugs. APS: ammonium persulfate. MBA: 4-sulfanylbutanimidamide.

**Figure 2.** Physical and chemical characterization of hydrogel composites. (a) Appearance of the materials, which are prepared with different concentrations of Prussian blue and the adsorption properties (unit = ug/ml). (b) Porous structure of formed composite photothermal hydrogels. (c) SEM microscopic morphology of Prussian blue nanoparticles. (d) UV absorption spectra of PB, PB@PAAm and TA-PB@PAAm. (e) Particle size distribution of Prussian blue nanoparticles. (f) Zeta potential of PB shows positive charge.

**Figure 3.** Physical and chemical characterization of TA-PB@PAAm composite hydrogels. (a) Swelling rate for different contents of composite photothermal hydrogels. (b) The preservation modulus G’ is always higher than the loss modulus G’’, which proves that the material has good crosslinking performance. (c) Photothermal effect curve for different PB concentrations. (d) Photothermal effect curve under different illumination intensities. (e) Curve of temperature changes after cyclic illumination of the hydrogels. (f) Drug release curve under alternating light.

**Figure 4.** CCK-8 results of NIH-3T3 cells co-cultured with composite photothermal hydrogels at different time points. (a–b) Absorbance in PAAm, PB@PAAm, TA@PAAm, TA-PB@PAAm with or without light. (c–d) Survival rate in PAAm, PB@PAAm, TA@PAAm, TA-PB@PAAm with or without light. Data are mean ± SD (n = 3, * p < 0.05, ** p < 0.01, *** p < 0.001, **** p < 0.0001, ns = no statistical difference).

**Figure 5.** In-vitro bactericidal test of composite photothermal hydrogel. (a) Bacteriostatic experiment of different hydrogels on *Staphylococcus aureus* and *Escherichia coli*. (b) The SEM result of the bacterial morphology through the treatment of different hydrogels on *Staphylococcus aureus* and *Escherichia coli*. Scale bar = 1μm. (c) Bacterial inhibition ring test results of different materials. Scale bar = 5mm.  (d) Quantitative results of the survival rate of *S. aureus*. (e) Quantitative results of survival rate of *E. coli*. (f) Quantitative results of the diameters of bacterial inhibition ring tests. Data are mean ± SD (n = 3, **** p < 0.0001).

**Figure 6.** Wound healing in SD rats. (a) Results of skin repair of infectious skin defect in animal model. Scale bar = 2 mm. (b) Quantitative results of the areas of wound healing. (c) In-vivo bacteriostatic experiments using different hydrogels on *Staphylococcus aureus*. (d) Quantitative results of the areas with a survival rate of *Staphylococcus aureus*. Data are mean ± SD (n = 3, * p < 0.05,** p < 0.01, **** p < 0.0001).

**Figure 7.** Histological staining evaluation. (a) Giemsa staining of infective skin defects shows the smallest number of bacteria for the L TA-PB@PAAm group. Black arrow represents bacteria. Scale bar = 20 μm. (b) Quantitative results of bacterial viability in Giemsa staining. (c) H&E staining showed skin repair results, neutrophils are fewer in the L PB@PAAm, N TA@PAAm, L TA@PAAm, NL TA-PB@PAAm, L TA-PB@PAAm groups. Black arrows represent inflammatory cells, red arrows represent newly formed vessels, and blue arrows represent newly formed hair follicles. Scale bar = 100 μm. (d) Quantitative results of inflammatory cells in H&E staining. (e) Masson’s trichrome staining shows the content of collagen are higher in the N TA@PAAm, L TA@PAAm, NL TA-PB@PAAm, L TA-PB@PAAm groups. (f) Quantitative results of collagen content in Masson’s trichrome staining. Data are mean ± SD (n = 3, * p < 0.05,** p < 0.01, **** p < 0.0001). Scale bar = 100 μm.

**Figure 8.** a H&E staining of heart, liver, spleen, lung and kidney. Scale bar = 100 μm. b Results of ALT. c Results of AST. d Results of BUN. e Results of CREA. Data are mean ± SD (n = 3, ns = no statistical difference).

**Figure S1.** FTIR characterization of different components.

**Figure S2.** XRD characterization of different components.

**Figure S3.** Results of tannic acid in UV-spectrum experiment. (a) Standard curve of tannic acid in different concentrations. (b) Absorbance profiles of tannic acid in different concentrations.

**Figure S4.** Determination of the 50% lethal dose of tannic acid and Prussian blue by CCK8 experiment. (a) Absorbance values of NIH-3T3 cells after intervention with different concentrations of tannic acid. (b) Absorbance values of different concentrations of Prussian blue after intervention in NIH-3T3 cells. Data are mean ± SD (n = 3,** p < 0.01, *** p < 0.001, **** p < 0.0001).

**Figure S5.** Bacterial inhibition ring test of Escherichia coli. There was no obvious bacterial inhibition ring around the hydrogel. Scale bar = 5 mm.
